# Age-Dependent Cortical Thinning of Peripheral Visual Field Representations in Primary Visual Cortex

**DOI:** 10.3389/fnagi.2016.00248

**Published:** 2016-10-25

**Authors:** Joseph C. Griffis, Wesley K. Burge, Kristina M. Visscher

**Affiliations:** ^1^Department of Psychology, University of Alabama at BirminghamBirmingham, AL, USA; ^2^Department of Neurobiology, University of Alabama at BirminghamBirmingham, AL, USA

**Keywords:** aging, visual cortex organization, primary visual cortex (V1), cortical thickness, structural MRI

## Abstract

The cerebral cortex changes throughout the lifespan, and the cortical gray matter in many brain regions becomes thinner with advancing age. Effects of aging on cortical thickness (CT) have been observed in many brain regions, including areas involved in basic perceptual functions such as processing visual inputs. An important property of early visual cortices is their topographic organization—the cortical structure of early visual areas forms a topographic map of retinal inputs. Primary visual cortex (V1) is considered to be the most basic cortical area in the visual processing hierarchy, and is topographically organized from posterior (central visual representation) to anterior (peripheral visual representation) along the calcarine sulcus. Some studies have reported strong age-dependent cortical thinning in portions of V1 that likely correspond to peripheral visual representations, while there is less evidence of substantial cortical thinning in central V1. However, the effect of aging on CT in V1 as a function of its topography has not been directly investigated. To address this gap in the literature, we estimated the CT of different eccentricity sectors in V1 using T1-weighted MRI scans acquired from groups of healthy younger and older adults, and then assessed whether between-group differences in V1 CT depended on cortical eccentricity. These analyses revealed age-dependent cortical thinning specific to peripheral visual field representations in anterior portions of V1, but did not provide evidence for age-dependent cortical thinning in other portions of V1. Additional analyses found similar effects when analyses were restricted to the gyral crown, sulcul depth and sulcul wall, indicating that these effects are not likely due to differences in gyral/sulcul contributions to our regions of interest (ROI). Importantly, this finding indicates that age-dependent changes in cortical structure may differ among functionally distinct zones within larger canonical cortical areas. Likely relationships to known age-related declines in visual performance are discussed to provide direction for future research in this area.

## Introduction

Aging is associated with changes in the structural properties of the cerebral cortex. Specifically, there is substantial evidence that aging leads to decreased thickness (thinning) of the cortical gray matter (Salat et al., 2004; Fjell et al., 2009; McGinnis et al., 2011). While it is generally accepted that widespread cortical thinning occurs as people age, there is some disagreement in the literature about the effects of aging on cortical thickness (CT) in early visual areas: several groups have reported decreased CT in primary visual cortex (V1) due to aging (Salat et al., 2004; Fjell et al., 2009; McGinnis et al., 2011), while others have not (Raz et al., 2004; Thambisetty et al., 2010; Lemaitre et al., 2012). These discrepancies might result, in part, from differences among studies in how V1 is defined. It is important to consider that cortex in V1 is organized as a topographic map of retinal inputs that progresses from foveal to peripheral retinotopic representations along the posterior-anterior dimension of the calcarine sulcus (Inoue, 1909; Fox et al., 1987; Engel et al., 1997), and it is therefore possible that different parts of this map may be differentially impacted by aging.

Importantly, portions of V1 that correspond to foveal vs. peripheral retinotopic representations differ in a variety of characteristics. These include differences in the cellular structure of retinal inputs to the lateral geniculate nucleus (LGN; Curcio and Allen, 1990; Dacey, 1993; Neitz et al., 2006), neuronal density (Collins et al., 2010), inter-regional connections (Hadjikhani and Tootell, 2000; Clavagnier et al., 2004; Palmer and Rosa, 2006), sensitivity to threatening stimuli (Bayle et al., 2009; Gomez et al., 2011), responses during cross-modal attention (Cate et al., 2009; Griffis et al., 2015), and modulation of activity by spatial attention (Roberts et al., 2007). Thus, it is reasonable to hypothesize that foveal vs. peripheral retinotopic representations in V1 might be differentially affected by processes such as aging. Indeed, several studies suggest that functional vision may decline differently for the central and peripheral fields (Drance et al., 1967; Haas et al., 1986), suggesting that age-related changes in the cortical structure of foveal representations in V1 (near the occipital pole) might be distinct from the developmental changes that occur further along the calcarine sulcus. Here, we examine how the CT of V1 at different retinotopic representations is affected by aging.

## Materials and Methods

### Participants

Thirty-two healthy younger adults (age range 19–32) and 41 older adults (age range 65–87) from the Birmingham area participated in this study. All participants did not have any psychiatric or neurological diagnoses at the time of the study. Participant demographics are shown in Table 1. Informed consent was obtained from all participants before proceeding with the experiment. All aspects of the study were approved by the University of Alabama at Birmingham Institutional Review Board.

**Table 1:** **Participant demographics**.

	Younger adults	Older adults
**Number of participants**	32	41
**Age (Mean/SD)**	25 (3.19)	71 (4.74)
**Sex (% male)**	53%	54%

### Anatomical MRI Acquisition

For each participant, we obtained a single 3D high-resolution MPRAGE T1-weighted anatomical scan using a 3 Tesla head-only Siemens Magnetom Allegra scanner. Repetition time (TR) = 2250 ms; echo time (TE) = 2.6 ms; inversion time (T1) = 900 ms; field of view (FOV[ap, fh, rl]) = 240 mm × 256 mm × 176 mm; slice gap, 0; 1.0 mm × 1.0 mm × 1.1 mm voxel size; flip angle (FA) = 9.

### Anatomical MRI Processing

Image processing (registration, segmentation and surface reconstruction) and CT estimation were performed using Freesurfer (version 5.3.0), a surface based analysis tool that estimates CT by measuring the distance between the boundary of gray/white mater and the pial surface (Dale et al., 1999; Fischl et al., 1999; Fischl and Dale, 2000). All cortical regions of interest (ROIs) were created using Freesurfer (version 5.3.0) on the cortical surface.

### V1 Regions of Interest

**Figure 1:**
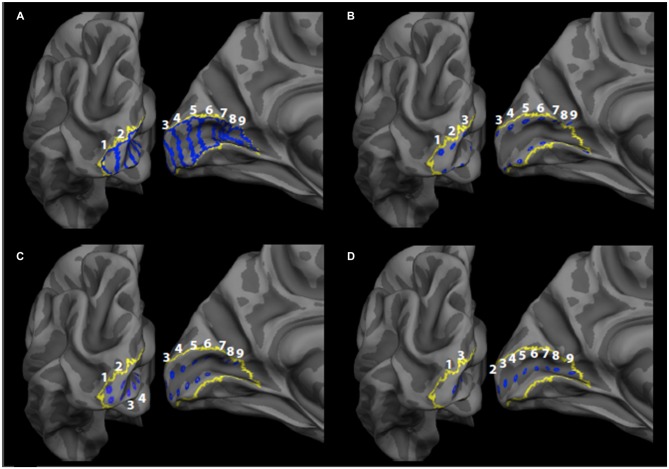
**Primary visual cortex (V1) regions of interest (ROIs).** The label boundaries (blue) for the **(A)** bar ROIs, **(B)** gyrus crown ROIs, **(C)** sulcus wall ROIs, and **(D)** sulcus depth ROIs are shown within the Freesurfer V1 label boundary (yellow) on the fsaverage brain. ROIs are numbered 1–9, such that one corresponds to the most posterior ROI (most central representation) and nine corresponds to the most anterior ROI (most peripheral representation).

For each hemisphere, a set of nine ROIs were defined on a flat-map of the occipital pole using the Freesurfer *fsaverage* brain as described in our previous publication (Burge et al., 2016). Each ROI was defined manually as a bar that extended across the dorsal-ventral axis of V1 and that was oriented perpendicular to the calcarine sulcus using the Freesurfer V1 label file as a guide (Figure 1A). The first 8 ROIs (moving anteriorly along the calcarine sulcus from the occipital pole) were each approximately 10 mm wide as estimated using the *plot_curv* function in *tksurfer*. An additional ninth ROI was also defined containing the remaining vertices in the space between the eighth ROI and the anterior-most end of the V1 label file (Figure 1A). While each ROI corresponded to different eccentricity sectors in V1, all of the bar ROIs contained the upper, middle and lower visual field representations in V1 (Inoue, 1909). Each of the manually defined ROIs were then transformed from the *fsaverage* space to the anatomical space of each participant, enabling consistent localization across participants. The mean surface area of the V1 bar ROIs was 309 mm^2^. Using a previously published probabilistic retinotopy template (Benson et al., 2012), we estimated the mean eccentricities (in degrees visual angle) of each ROI as <1°, 1.34°, 2.2°, 4.1°, 7.3°, 14.1°, 25.5°, 40.0° and 63.3°, respectively. It should be noted that the eccentricity of the first ROI is less precisely defined because the lower limit of the probabilistic template is 1° visual angle, due to the difficulty of retinotopic mapping at the foveal confluence (Benson et al., 2012).

### Controlling for Ratio of Gyrus Tissue to Sulcus Tissue

CT at the depth of sulci in the brain has been found to be, in general, less than at the gyral crowns (Fischl and Dale, 2000). To exclude the possibility that the effects observed for the previously described bar ROIs might be due to disproportionate representations of the sulcal depth or gyral crowns in a given ROI, we created additional ROIs that allowed us to separately measure CT at each location separately. Each ROI was created as a grouping of vertices on the cortical surface (Figures 1B–D) within each bar ROI. The additional ROIs consisted of ROIs in the *gyrus crown* that were located on the V1 side of the V1/V2 border on both the upper and lower banks of the calcarine sulcus (Figure 1B), ROIs in the *sulcus wall* that were located halfway between the gyrus crown and the depth of the sulcus for both the upper and lower banks of the calcarine sulcus (Figure 1C), and ROIs in the *sulcus depth* (Figure 1D). Each ROI was defined in Freesurfer by manually selecting a vertex within the initial set of bar ROIs and creating a Freesurfer “label.” Each single vertex was then dilated using the FreeSurfer “Dilate Label” function, which dilates the original vertex label to include neighboring vertices. Each label was dilated a total of three times. The area of each of these regions was roughly 20 mm^2^, and each ROI was spaced roughly 10 mm apart. The ROIs were then transformed from the *fsaverage* brain to each participant’s anatomical space, as described above for the bar ROIs.

### Statistical Analyses

To evaluate the effects of age on CT across different eccentricities in V1, we performed mixed measures analysis of variances (ANOVAs) with a between-subjects factor of group (two levels—younger adults vs. older adults) and a within-subjects factor of eccentricity (nine levels—one for each ROI). Identical ANOVAs were performed for each set of ROIs. Interaction effects were of primary interest, as they would indicate that differences in CT between the groups depended on eccentricity. Interactions were considered significant if *p*-values for the interaction term were not greater than 0.05 (Bonferroni-Holm corrected across four ANOVAs). Sphericity violations were corrected using Greenhouse-Geisser adjustments to the number of degrees of freedom (Abdi, 2010). *Post hoc* independent samples *t*-tests were performed to identify ROIs showing significant differences between groups as follow-ups to significant interaction effects, and were considered significant if *p*-values were not greater than 0.05 (Bonferroni-Holm corrected across nine ROIs).

## Results

### Bar ROIs

**Figure 2:**
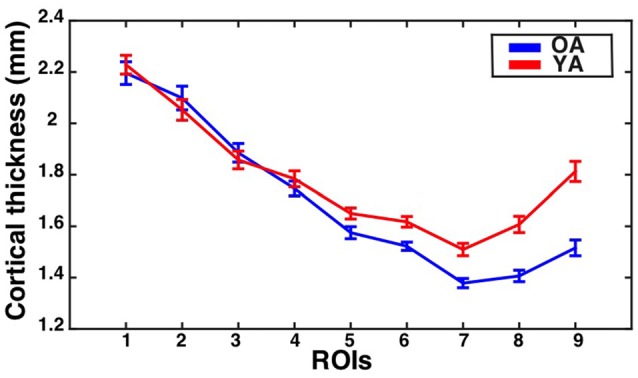
**Cortical thickness (CT) for the V1 bar ROIs as a function of eccentricity for younger and older adults.** Mean ± standard error of the mean for CT is shown for each group at each bar ROI. Values for older adults are plotted in blue, while values for younger adults are plotted in red.

The assumption of sphericity was violated for the repeated measures factor (Greenhouse-Geisser epsilon = 0.53, *p* < 0.001). Therefore, Greenhouse-Geisser adjustment was performed on the degrees of freedom to account for this violation. The analysis revealed a significant eccentricity by group interaction effect (*F*_(4.2,298.6)_ = 8.28, corrected *p* < 0.001, partial eta-squared = 0.10), a main effect of eccentricity (*F*_(4.2,298.65)_ = 189.64, *p* < 0.001, partial eta-squared = 0.73), and a main effect of group (*F*_(1,71)_ = 10.15, *p* = 0.002, partial eta-squared = 0.13). To identify regions showing significant differences in CT between the groups, follow-up independent samples *t*-tests were performed at each ROI and corrected using the Bonferonni-Holm procedure to control the family-wise error rate at 0.05. This revealed that the older adults had significantly thinner cortex than young adults in ROI 6 (*t*_(71)_ = −3.67, corrected *p* = 0.003), ROI 7 (*t*_(71)_ = −4.41, corrected *p* < 0.001), ROI 8 (*t*_(71)_ = −5.31, corrected *p* < 0.001), and ROI 9 (*t*_(71)_ = −6.03, corrected *p* < 0.001). Results are illustrated in Figure 2.

### Gyrus Crown ROIs

**Figure 3:**
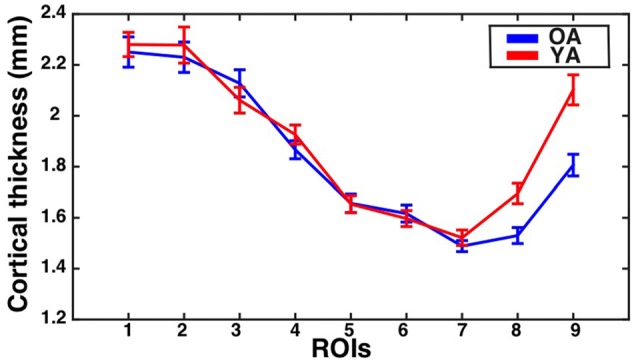
**CT for the V1 gyrus crown ROIs as a function of eccentricity for younger and older adults.** Mean ± standard error of the mean for CT is shown for each group at each gyrus crown ROI. Values for older adults are plotted in blue, while values for younger adults are plotted in red.

The assumption of sphericity was violated for the repeated measures factor (Greenhouse-Geisser epsilon = 0.67, *p* < 0.001). Greenhouse-Geisser adjustment was performed on the degrees of freedom to account for this violation. The analysis revealed a significant eccentricity by group interaction effect (*F*_(5.35,379.49)_ = 3.35, corrected *p* = 0.01, partial eta-squared = 0.05), a main effect of eccentricity (*F*_(5.35,379.49)_ = 95.72, *p* < 0.001, partial eta-squared = 0.57), and a main effect of group (*F*_(1,71)_ = 3.82*, p* = 0.05, partial eta-squared = 0.05). To identify regions showing significant differences in CT between the groups, follow-up independent samples *t*-tests were performed at each ROI and corrected using the Bonferonni-Holm procedure to control the family-wise error rate at 0.05. This revealed that the older adults had significantly thinner cortex than young adults in ROI 8 (*t*_(71)_ = −3.30, corrected *p* = 0.01) and ROI 9 (*t*_(71)_ = −4.15, corrected *p* < 0.001). Results are illustrated in Figure 3.

### Sulcus Depth ROIs

**Figure 4:**
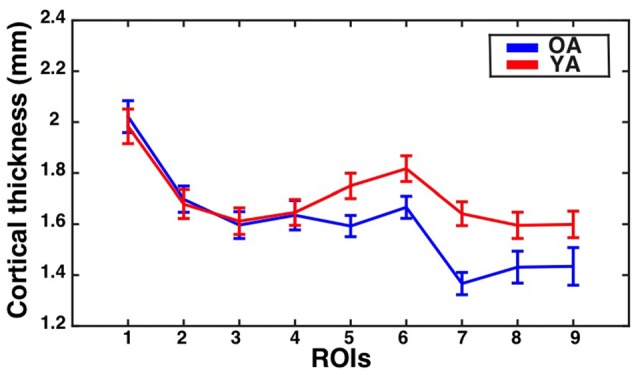
**CT for the V1 sulcus depth ROIs as a function of eccentricity for younger and older adults.** Mean ± standard error of the mean for CT is shown for each group at each sulcus depth ROI. Values for older adults are plotted in blue, while values for younger adults are plotted in red.

The assumption of sphericity was violated for the repeated measures factor (Greenhouse-Geisser epsilon = 0.75, *p* < 0.001). Greenhouse-Geisser adjustment was performed on the degrees of freedom to account for this violation. The analysis revealed a significant eccentricity by group interaction effect (*F*_(5.96,423.44)_ = 2.11, corrected *p* = 0.05, partial eta-squared = 0.03), a main effect of eccentricity (*F*_(5.96,423.44)_ = 17.63, *p* < 0.001, partial eta-squared = 0.20) and a main effect of group (*F*_(1,71)_ = 7.61, *p* < 0.001, partial eta-squared = 0.10). To identify regions showing significant differences in CT between the groups, follow-up independent samples *t*-tests were performed at each ROI and corrected using the Bonferonni-Holm procedure to control the family-wise error rate at 0.05. This revealed that the older adults had significantly thinner cortex than young adults in ROI 7 (*t*_(71)_ = −4.24, corrected *p* < 0.001). Results are illustrated in Figure 4.

### Sulcus Wall ROIs

**Figure 5:**
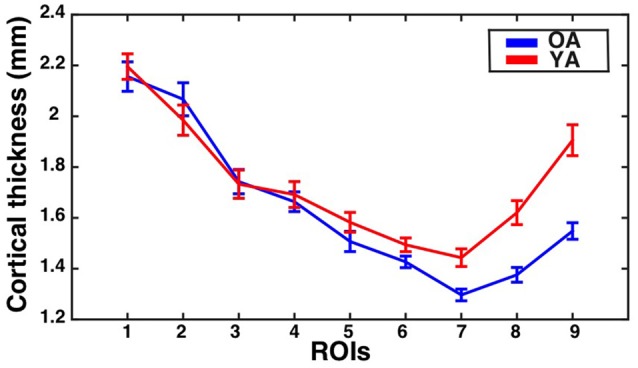
**CT for the V1 sulcus wall ROIs as a function of eccentricity for younger and older adults.** Mean ± standard error of the mean for CT is shown for each group at each sulcal wall ROI. Values for older adults are plotted in blue, while values for younger adults are plotted in red.

The assumption of sphericity was violated for the repeated measures factor (Greenhouse-Geisser epsilon = 0.67*, p* < 0.001). Greenhouse-Geisser adjustment was performed on the degrees of freedom to account for this violation. The analysis revealed a significant eccentricity by group interaction effect (*F*_(5.36,380.64)_ = 5.60, corrected *p* = 0.002, partial eta-squared = 0.07), a main effect of eccentricity (*F*_(5.36,380.64)_ = 87.38, *p* < 0.001, partial eta-squared = 0.55), and a main effect of group (*F*_(1,71)_ = 7.58, *p* = 0.007, partial eta-squared = 0.10). To identify regions showing significant differences in CT between the groups, follow-up independent samples *t*-tests were performed at each ROI and corrected using the Bonferonni-Holm procedure to control the family-wise error rate at 0.05. This revealed that the older adults had significantly thinner cortex than young adults in ROI 7 (*t*_(71)_ = −3.66, corrected *p* = 0.003), ROI 8 (*t*_(71)_ = −4.62, corrected *p* < 0.001), and ROI 9 (*t*_(71)_ = −5.49, corrected *p* < 0.001). Results are illustrated in Figure 5.

## Discussion

In the current study, we assessed the effect of aging on CT in human V1. Specifically, we investigated whether age-dependent changes in the CT of V1 might differ among portions of V1 that represent different visual eccentricities. Our analyses revealed that while the regions of V1 corresponding to peripheral visual representations had significantly thinner cortex in older adults than in younger adults, the regions of V1 corresponding to central visual representations did not show significant differences in CT between groups. Additionally, our results indicate that these effects are unlikely to be driven by differences in gyral/sulcul contributions to central vs. peripheral eccentricity sectors. Together, our results suggest that age-dependent cortical thinning in human V1 is specific to portions that represent peripheral visual space.

Several previous whole-brain studies of age-related changes in CT have found consistent evidence for age-dependent thinning in early visual areas including V1 (Salat et al., 2004; Fjell et al., 2009; McGinnis et al., 2011). In contrast, a previous study using manually defined ROIs corresponding to only a portion of V1 did not find strong evidence for cortical thinning in V1 (Raz et al., 2004). Other recent studies have not found substantial age-related thinning in V1 when assessing age-related changes in the CT of V1 as a whole (Thambisetty et al., 2010; Lemaitre et al., 2012). The current article suggests an explanation of these divergent findings: age-dependent cortical thinning in V1 primarily occurs in areas corresponding to peripheral visual field representations. Here, we performed a comprehensive analysis assessing the effects of aging on CT in anatomically defined sub-regions of V1 that correspond to different representations of visual space. The results of our analyses provide evidence for age-related cortical thinning that is specific to anterior portions of the calcarine sulcus (corresponding to representations of peripheral visual space), but do not provide support for age-related cortical thinning in posterior portions of the calcarine sulcus (corresponding to representations of central visual space). These results confirm and build upon previous reports of age-dependent cortical thinning in V1 (Salat et al., 2004; Fjell et al., 2009; McGinnis et al., 2011), by indicating that age-dependent changes in cortical structure may be selective to specific, functionally distinct sub-regions of larger anatomical areas.

As mentioned previously, the other studies that have found evidence for age-dependent thinning in V1 have used whole-brain approaches that separately model the effects of aging at each surface vertex (Salat et al., 2004; Fjell et al., 2009; McGinnis et al., 2011). Interestingly, the calcarine sulcus ROI identified by Salat et al. (2004) by their whole-brain analysis as showing maximal age-dependent cortical thinning consisted primarily of anterior portions of the calcarine sulcus, consistent with our results here, though that analysis did not directly compare central to peripheral visual cortex. In contrast, the current study directly assessed whether the effects of aging on CT varied across central and peripheral parts of V1. Similarly, the strongest aging effects in visual cortex identified by the whole-brain analyses reported by McGinnis et al. (2011) were also primarily located in more anterior calcarine regions. In contrast, the V1 ROI employed by Raz et al. (2004) likely consisted primarily of the cortex within the sulcus of regions corresponding to the mid-periphery, although it is difficult to discern the precise extent of their ROI based on the description/figure provided in the report. Another recent study that analyzed the effects of aging on the CT of V1 as a whole only identified substantial aging effects when global decreases in CT were not modeled as a control for global aging effects (Lemaitre et al., 2012). The current study, in a sense, uses V1 as its own control by directly comparing aging effects across different eccentricity sectors, making our results unlikely to be influenced by global aging effects. Indeed, the results of our analyses suggest that while some age-dependent cortical thinning may be present in middle portions of the calcarine sulcus, the most substantial thinning is observed in more anterior regions (Figures 2–4), and this effect may not be observed by studies that estimate the average effect of aging on the thickness of V1 as a whole.

While the present study did not incorporate behavioral measurements, it is worth discussing that the effective use of vision declines with healthy aging. For example, measures of low-level vision such as acuity and contrast sensitivity decline with age (Sekuler and Sekuler, 2000; Owsley, 2011). Central and peripheral fields show similar declines with age in some low level measures (Haegerstrom-Portnoy et al., 1999), but other metrics appear to show that peripheral vision may be more starkly influenced by age than central vision (Drance et al., 1967; Haas et al., 1986). While some of these declines in vision come from aging of the anterior eye, for example yellowing of the lens or decreased pupil size, age-related changes in vision also stem from a neural source (Johnson et al., 1989).

Standard acuity tests of visual function appear to underestimate the degree of difficulty that older adults have in doing everyday activities that require peripheral vision (e.g., driving; Ball et al., 1990). However, somewhat more complex vision tests appear to better predict behaviors on everyday tasks like driving (Ball et al., 1993; Owsley et al., 1998). These measures incorporate peripheral stimuli and performance appears to decline steeply with age (Ball and Owsley, 1993). Several studies using different approaches have shown that the ability to identify a stimulus in the periphery in the presence of distracting information in the center declines with age (Scialfa et al., 1987; Ball and Owsley, 1993; Haegerstrom-Portnoy et al., 1999; Sekuler et al., 2000). Thus, previously reported data show that performance on complex visual tasks using peripheral visual information decline with age. Together with the data from the current manuscript, this suggests that age-related declines in complex visual tasks using peripheral vision might result from age-related changes in the neural representations of peripheral vision.

A great deal of work examining simultaneous central and peripheral stimuli has used a task called the “Useful Field of View” Task. It is interesting to note that early descriptions of this work interpreted age-associated declines in performance in terms of declines of peripheral vision (e.g., Ball et al., 1988). This mirrors an early work which suggested that the size of the visual field shrinks with age (Drance et al., 1967; Haas et al., 1986). However, more recent articles have interpreted these declines in Useful Field of View performance as resulting from declines in Speed of Processing, the speed with which a participant can take in and process information from the full visual field (e.g., Ball et al., 2007). The two interpretations share many factors—for example, peripheral information must be processed quickly for good speed of processing performance. Our data are particularly interesting because they imply that the brain regions that are specific to peripheral information are selectively thinned through the aging process. Future work should examine the degree to which this selective thinning relates to individual differences in general visual behavior such as speed of processing as well as measures of low level vision in the peripheral vs. central field.

Participants with adult-onset central vision loss are forced to use peripheral vision for daily tasks. These participants have thicker cortex compared to normally sighted controls in peripheral parts of V1 (corresponding to the parts of the visual field with increased attentive use; Burge et al., 2016). This suggests that the thickness of cortex is use-dependent, and appears to be plastic, even in adults. The majority of attention-demanding tasks that a person performs in a typical day involve central vision: recognizing faces, tasks requiring fine hand-eye coordination, reading, etc. One explanation for our data is that a lifetime of experience attending primarily to central vision, but often ignoring peripheral vision might contribute to thinning in the parts of cortex associated with peripheral vision in older age. Further research is needed to directly test this hypothesis by investigating the relationships between the age-dependent thinning of peripheral V1 and age-dependent changes in peripheral visual function.

It is also important to note that these data were not initially collected with the explicit goal of testing age related differences. Our groups were not matched on race or education. These results should be interpreted with the cross sectional design of this study in mind. Nonetheless, the results indicate that future studies should consider the possibility of non-uniform aging effects in cortical areas such as V1 that contain distinct functional subdivisions.

## Author Contributions

JCG, WKB and KMV contributed to the study conceptualization/design. WKB and KMV contributed to data collection. JCG, WKB and KMV contributed to data analysis and interpretation. JCG and KMV wrote the manuscript.

## Conflict of Interest Statement

The authors declare that the research was conducted in the absence of any commercial or financial relationships that could be construed as a potential conflict of interest.
